# Variations in the Volatile Compositions of *Curcuma* Species

**DOI:** 10.3390/foods8020053

**Published:** 2019-02-02

**Authors:** Noura S. Dosoky, Prabodh Satyal, William N. Setzer

**Affiliations:** 1 Aromatic Plant Research Center, 230 N 1200 E, Suite 100, Lehi, UT 84043, USA; ndosoky@aromaticplant.org; 2 Department of Chemistry, University of Alabama in Huntsville, Huntsville, AL 35899, USA; psatyal@aromaticplant.org

**Keywords:** *Curcuma aeruginosa*, *Curcuma longa*, *Curcuma zedoaria*, *Curcuma aromatica*, rhizome essential oils

## Abstract

*Curcuma* species have been cultivated in tropical and subtropical regions in Asia, Australia, and South America for culinary as well as medicinal applications. The biological activities of *Curcuma* have been attributed to the non-volatile curcuminoids as well as to volatile terpenoids. *Curcuma* essential oils have demonstrated a wide variety of pharmacological properties. The objective of this work was to examine the variation in the compositions of *Curcuma* rhizome essential oils. In this work, the volatile oils from *C. longa* and *C. zedoaria* were obtained and analyzed by gas chromatography-mass spectrometry. The chemical compositions of *C. longa* and *C. zedoaria* essential oils, including those reported in the literature, were analyzed by hierarchical cluster analysis. In addition, cluster analyses of the chemical compositions of *C. aromatica* and *C. aeruginosa* from the literature were also carried out. *Curcuma longa* volatiles were dominated by α-turmerone, curlone, *ar*-turmerone, β-sesquiphellandrene, α-zingiberene, germacrone, terpinolene, *ar*-curcumene, and α-phellandrene and showed four distinct chemical clusters. *C. zedoaria* rhizome oil contained 1,8-cineole, curzerenone/*epi*-curzerenone, α-copaene, camphor, β-caryophyllene, elemol, germacrone, curzerene, and β-elemene and showed two different chemical types. *C. aromatica* had three clearly defined clusters, and *C. aeruginosa* had three types.

## 1. Introduction

The genus *Curcuma* L. (Zingiberaceae) consists of about 93–100 species of perennial rhizomatous herbs that originated in tropical and subtropical regions of Asia, Australia, and South America [1]. Many of these species are extensively grown on a very large scale in India, Pakistan, Indonesia, Malaysia, Bangladesh, Nepal, and Thailand [2]. *Curcuma* species are greatly valued for their medicinal properties. For hundreds of years, members of *Curcuma* have been used in traditional medicine for treating respiratory complaints, pain, digestive disorders, inflammatory conditions, wounds, hypercholesterolemia, hypertension, hematologic and circulation abnormalities, infectious diseases, and cancer prevention, among others [3,4,5]. They are also important sources of flavoring and coloring agents, cosmetics, perfumes, and ornamental plants [5,6]. *Curcuma* species possess a variety of pharmacological activities including anti-inflammatory, antiproliferative, anticancer, hypoglycemic, anti-hyperlipidemic, antiatherosclerotic, neuroprotective, hepatoprotective, anti-diarrheal, carminative, diuretic, antirheumatic, anticonvulsant, hypotensive, antioxidant, insecticidal, larvicidal, antimicrobial, antiviral, antivenomous, anti-thrombotic, and antityrosinase activities [7,8,9,10,11,12,13,14,15].

The rhizome, which contains a variety of terpenoids, flavonoids, and phenylpropanoids [16], is the most extensively used part of the plant [17]. Several studies indicated that the bioactive ingredients of *Curcuma* rhizome are the non-volatile curcuminoids (curcumin, demethoxycurcumin, and bisdemethoxycurcumin) and the volatile oil (sesquiterpenoids and monoterpenoids) [14,18]. Curcumin, the most active curcuminoid in turmeric rhizome, has anticancer [19], anti-inflammatory [20], antioxidant [21], antibacterial, anti-fungal [22], analgesic, digestive, antidepressant [23], and hypoglycemic [23] properties and has shown potential against cardiovascular diseases [24] and Alzheimer’s disease [25]. *Curcuma* essential oil (EO) is often extracted by distillation of the fresh or dry rhizome [26], or by supercritical fluid extraction [27]. Generally, the *Curcuma* oils are made up of sesquiterpenoids and monoterpenoids [5]. There is a great variation in the literature on *Curcuma* EO due to differences in the genotype, edaphic factors, climate, time of harvest, extraction, and analysis methods [28,29,30]. Around 31 *Curcuma* species have been studied of which *C. longa* (turmeric) and *C. zedoaria* (zedoary) are the most extensively investigated [5]. The current study was conducted to investigate the composition and different chemotypes of the rhizome essential oils of *C. longa* L., *C. aromatica* Salisb., *C. zedoaria* (Christm.) Roscoe, and *C. aeruginosa* Roxb. from collections from different geographical origins.

## 2. Materials and Methods

### 2.1. Volatile Oils

Volatile oils from commercial suppliers were obtained from the collections of the Aromatic Plant Research Center (APRC, Lehi, UT, USA). A total of 33 *Curcuma longa* (turmeric) rhizome oils from the APRC collection, including 24 hydro- or steam-distilled essential oils, five supercritical CO_2_ extracts, and four oils of unknown origin or extraction method, were analyzed by gas chromatography–mass spectrometry (GC-MS).

### 2.2. Gas Chromatographic-Mass Spectral Analysis

The essential oils obtained from APRC were analyzed by gas chromatography-mass spectrometry (GC-MS) using a Shimadzu GCMS-QP2010 Ultra operated in the electron impact (EI) mode (electron energy = 70 eV), scan range = 40–400 atomic mass units, scan rate = 3.0 scans/s, and GC-MS solution software (Shimadzu Scientific Instruments, Columbia, MD, USA). The GC column was a ZB-5 fused silica capillary column with a (5% phenyl)-polymethylsiloxane stationary phase and a film thickness of 0.25 μm, a length of 30 m, and an internal diameter of 0.25 mm (Phenomenex, Torrance, CA, USA). The carrier gas was helium with a column head pressure of 552 kPa and flow rate of 1.37 mL/min. The injector temperature was 250 °C and the ion source temperature was 200 °C. The GC oven temperature was programmed for 50 °C initial temperature, then temperature was increased at a rate of 2 °C/min to 260 °C. A 7% *w*/*v* solution of the sample was prepared in dichloromethane and 0.1 μL was injected with a splitting mode (30:1). Identification of the oil components was based on their retention indices determined by reference to a homologous series of n-alkanes, and by comparison of their mass spectral fragmentation patterns with those reported in the literature [31] and our own in-house library [32].

### 2.3. Hierarchical Cluster Analysis

The chemical compositions of the *Curcuma* oils obtained from this work as well as the published literature were used in the cluster analysis. The essential oil compositions were treated as operational taxonomic units (OTUs), and the concentrations (percentages) of the major components (*C. longa*: α-phellandrene, *p*-cymene, 1,8-cineole, terpinolene, *ar*-curcumene, α-zingiberene, β-bisabolene, β-sesquiphellandrene, *ar*-turmerone (= dehydroturmerone), α-turmerone, germacrone, curlone (= β-turmerone), (6*S*,7*R*)-bisabolone, and (*E*)-α-atlantone; *C. zedoaria*: 1,8-cineole, camphor, α-copaene, β-elemene, β-caryophyllene, *ar*-curcumene, zingiberene, curzerene, germacrene B, β-sesquiphellandrene, curzerenone/*epi*-curzerenone, and germacrone; *C. aromatica*: α-pinene, camphene, 1,8-cineole, camphor, isoborneol, borneol, β-elemene, *ar*-curcumene, curzerene, β-curcumene, curzerenone, germacrone, xanthorrhizol, and curdione (= 1(10)-germacrene-5,8-dione; *C. aeruginosa*: camphene, β-pinene, 1,8-cineole, camphor, isoborneol, borneol, β-elemene, β-farnesene, zingiberene, curzerene, germacrene B, curzerenone, β-eudesmol, germacrone, and curcumenol) were used to determine the chemical associations between the essential oils using agglomerative hierarchical cluster (AHC) analysis using XLSTAT Premium, version 2018.5.53172 (Addinsoft, Paris, France). Dissimilarity was determined using Euclidean distance, and clustering was defined using Ward’s method.

## 3. Results and Discussion

Essential oils from the *Curcuma* species were obtained from a collection of oils from commercial sources deposited with the Aromatic Plant Research Center (APRC). *Curcuma* species are known for producing an array of volatile sesquiterpenes, monoterpenes, and other aromatic compounds [5,15]. Hundreds of compounds have been identified from the turmeric oils, however, the major components were α-turmerone (12.6–44.5%), curlone (9.1–37.8%), *ar*-turmerone (12.2–36.6%), β-sesquiphellandrene (5.0–14.6%), α-zingiberene (5.0–12.8%), germacrone (10.3–11.1%), terpinolene (10.0–10.2%), *ar*-curcumene (5.5–9.8%), and α-phellandrene (5.0–6.7%) (Table 1). Interestingly, Brazilian turmeric EO samples showed (*Z*)-γ-atlantone, *ar*-turmerone, and (*E*)-γ-atlantone as the main constituents [33], while a sample from north central Nigeria had β-bisabolene, (*E*)-β-ocimene, β-myrcene, 1,8-cineole, α-thujene, α-phellandrene, limonene, zingiberene, and β-sesquiphellandrene [34]. Turmeric oils of Sri Lanka and São Tomé e Principe origins had α-phellandrene, α-turmerone, 1,8-cineole, *p*-cymene, *ar*-turmerone, β-turmerone, and terpinolene as the major components [10,35].

The rhizome of *Curcuma aromatica* (commonly known as wild turmeric) is a traditional medicine used to alleviate pain, eliminate blood stasis, and slow ageing [36]. The Japanese *C. aromatica* oil was reported to have curdione (32.2–44.0%), 1,8-cineole (7.5–25.3%), and germacrone (4.6–9.6%) [37], while a sample from Thailand contained camphor (26.9%), *ar*-curcumene (23.2%), and xanthorrhizol (18.7%) as the main components [38]. Indian samples of *C. aromatica* had camphor (18.2–48.3%), β-curcumene (28.4–31.4%), *ar*-curcumene (22.1–24.1%), xanthorrhizol (4.8–16.2%), 1,8-cineole (5.5–15.9%), isoborneol (8.2–12.2%), curzerenone (5.5–11.0%), germacrone (4.9–10.6%), camphene (7.4–10.2%), curdione (4.8–8.0%), borneol (4.9–8.2%), β-elemene (7.5%), curzerene (4.6–6.0%), α-pinene (5.7–5.9%), and terpinolene (5.2%) [15,37,39,40,41,42] (Table 2).

Zedoary (*Curcuma zedoaria*) rhizome is also called “white turmeric” because of its similarity to ginger from the outside and to turmeric from the inside. Zedoary EO is generally made of sesquiterpenoids (80–85%) and monoterpenoids (15–20%). The major components of *C. zedoaria* rhizome oil are 1,8-cineole (7.0–38.4%), curzerenone/*epi*-curzerenone (20.9–29.4%), α-copaene (17.4%), camphor (8.6–8.8%), β-caryophyllene (8.8%), elemol (6.8%), germacrone (6.7%), curzerene (5.9%), and β-elemene (5.5%) (Table 3). The main components of *C. zedoaria* rhizome oil reported in the literature were curzerenone/*epi*-curzerenone (19.0–31.6%), curzerene (8.0%), *ar*-curcumene (12.1%), zingiberene (12.0%), germacrone (10.8%), camphor (10.3%), β-sesquiphellandrene (9.8%), and germacrene B (6.0%) [15,43].

*Curcuma aeruginosa* (also known as “black curcuma”) is characterized by its distinctive ginger-like scent [44]. The volatile oil of *C. aeruginosa* is known to contain relatively equal amounts of monoterpenes and sesquiterpenes. Two black turmeric samples from Malaysia had curzerenone (24.6–30.4%), 1,8-cineole (11.2–25.2%), camphor (6.8–10.5%), and curcumenol (5.6%) [45,46], while from India the oil was dominated by curcumenol (38.7%) and β-pinene (27.5%) [15] (Table 4). A *C. aeruginosa* oil sample from Thailand was dominated by curzerenone (41.6%) followed by 1,8-cineole (9.6%) and β-pinene (7.7%) [38], whereas another sample had camphor (29.4%), germacrone (21.2%), isoborneol (7.3%), germacrene B (5.2%), and curzerene (4.8%) [4].

**Figure 1:**
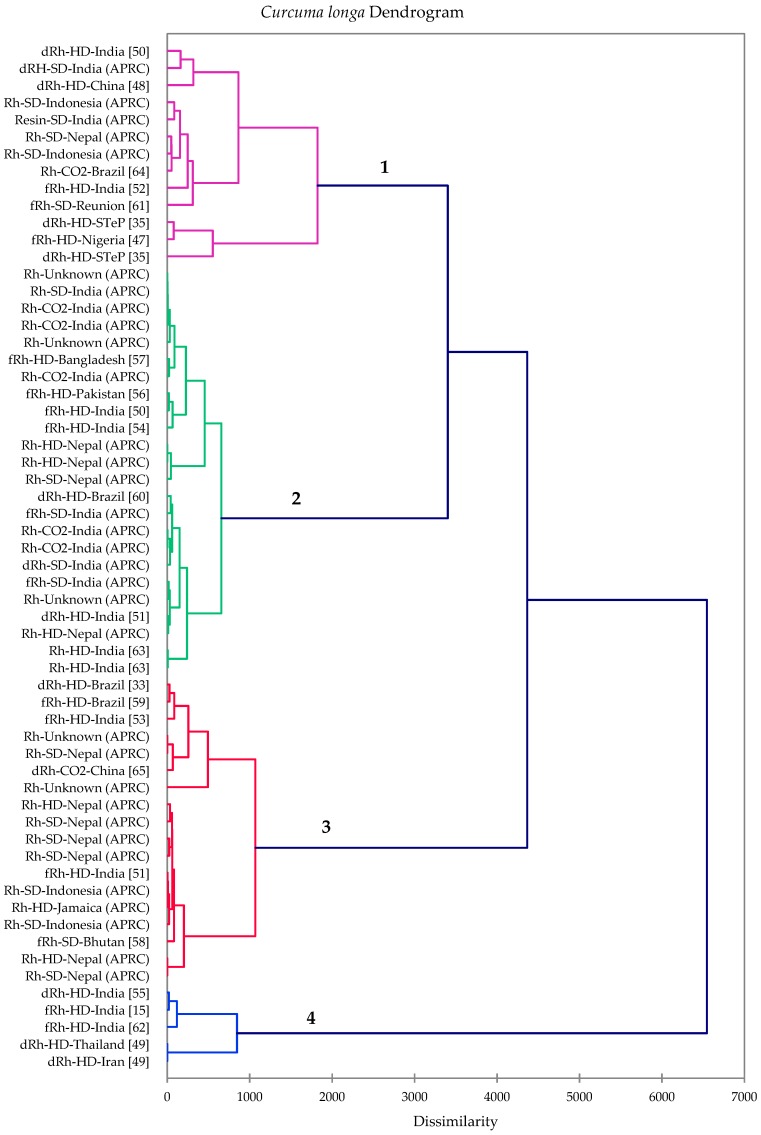
Dendrogram obtained from the agglomerative hierarchical cluster analysis of 60 *Curcuma longa* volatile oil samples.

A hierarchical cluster analysis was carried out based on the *C. longa* essential oil compositions. For comparison, we included *C. longa* rhizome oils that were reported in the literature in this analysis, including 23 steam- or hydrodistilled essential oils and two supercritical CO_2_ extracts (Table 5). Although *C. longa* rhizome oils were all rich in *ar*-turmerone, α-turmerone, and β-turmerone, the cluster analysis revealed four clearly defined clusters based on the relative concentrations of these major components (Figure 1). The cluster centroids of the major components of *C. longa* rhizome oils are summarized in Table 6, illustrating the chemical differences in the four clusters. Cluster 2 was the largest, representing 21 samples dominated by the turmerones (particularly *ar*-turmerone). Cluster 1 represents samples with relatively large concentrations of components other than turmerones; therefore, lower concentrations of turmerones. The third cluster was also a large cluster, representing 15 samples dominated by the turmerones (predominantly α-turmerone). The fourth cluster had very large concentrations of *ar*-turmerone.

**Figure 2:**
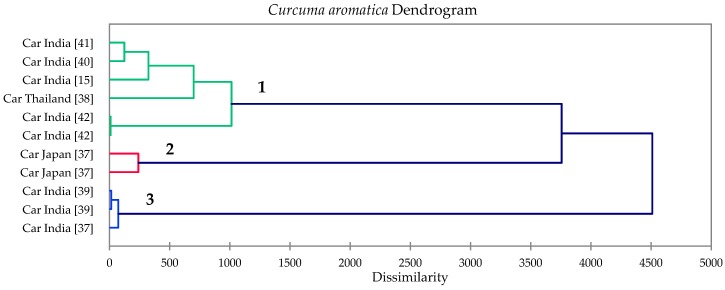
Dendrogram obtained from the agglomerative hierarchical cluster analysis of eight *Curcuma aromatica* essential oil samples.

Hierarchical cluster analysis of *C. aromatica* essential oils clearly identified three clusters based on dissimilarity (Figure 2). Cluster 1 had a relatively high camphor concentration, represented by the *C. aromatica* EO sample from Thailand [38]; cluster 2 was dominated by curdione followed by 1,8-cineole, represented by two samples from Japan [37]; and cluster 3 represents samples with large concentrations of *ar*-curcumene and β-curcumene [15,37,39,40,41,42]. Table 7 summarizes the cluster centroids of the major components of *C. aromatica* rhizome oils.

**Figure 3:**
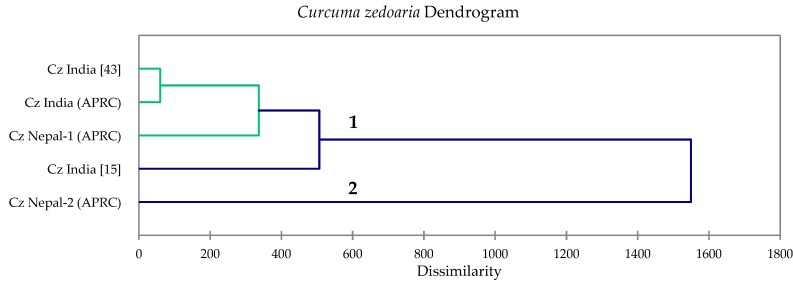
Dendrogram obtained from the agglomerative hierarchical cluster analysis of five *Curcuma zedoaria* essential oil samples.

For *C. zedoaria* essential oils, the cluster analysis showed two clusters based on dissimilarity (Figure 3): (1) a cluster dominated by curzerenone/*epi*-curzerenone followed by camphor, germacrone, 1,8-cineole, and α-copaene; and (2) a cluster represented by a single sample with very large concentrations of 1,8-cineole. The cluster centroids of the main constituents of *C. zedoaria* rhizome oils are summarized in Table 8.

**Figure 4:**
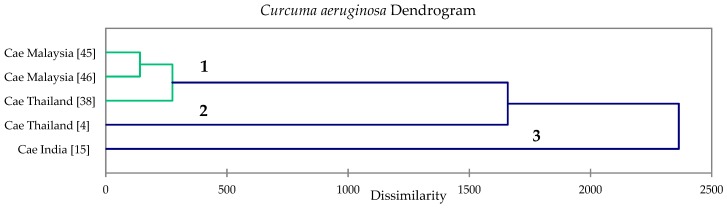
Dendrogram obtained from the agglomerative hierarchical cluster analysis of five *Curcuma aeruginosa* essential oil compositions.

*Curcuma aeruginosa* essential oils showed three classes in hierarchical cluster analysis based on dissimilarity (Figure 4): (1) a camphor/germacrone rich cluster with large concentrations of isoborneol, curzerene, and germacrone B; (2) a curcumenol/β-pinene rich cluster; and (3) a curzerenone/1,8-cineole cluster. Table 9 summarizes the concentrations of cluster centroids of the major components of *C. aeruginosa* rhizome oils. Although there are only five essential oil samples of *C. zedoaria* and *C. aeruginosa*, which is too few to give a comprehensive chemotaxonomic representation of these species, this analysis does provide initial insights into the potential chemotypes.

## 4. Conclusions

The rhizome essential oils of *Curcuma longa*, *C. aromatica*, *C. zedoaria*, and *C. aeruginosa* from the APRC collection, compared to the published literature, were analyzed by GC-MS. α-Turmerone, curlone, *ar*-turmerone, β-sesquiphellandrene, α-zingiberene, germacrone, terpinolene, *ar*-curcumene, and α-phellandrene were the major components of *C. longa. C. zedoaria* rhizome oil contained 1,8-cineole, curzerenone/*epi*-curzerenone, α-copaene, camphor, β-caryophyllene, elemol, germacrone, curzerene, and β-elemene. The cluster analysis revealed four clearly defined clusters for *C. longa*, three clusters for *C. aromatica* and *C. aeruginosa*, and two types for *C. zedoaria*.

In the case of *C. longa*, there are no apparent correlations based on extraction method (steam distillation, hydrodistillation, or supercritical CO_2_ extraction) or country or region of origin. Furthermore, the differences between the clusters are not that great, and therefore, the clusters do not likely represent distinct chemotypes but rather just reflect the chemical variation within each species. The data do provide a baseline for comparison of *C. longa* rhizome oils, however. These are important points when considering sources of either essential oils or rhizomes. There are still too few data to draw conclusions about the possible chemotypes of *C. aromatica*, *C. aeruginosa*, or *C. zedoaria*; more data are required.

## Figures and Tables

**Table 1:** Chemical compositions (major components) of *Curcuma longa* rhizome volatile oils.

Sample	α-Phellandrene	*p*-Cymene	1,8-Cineole	Terpinolene	*ar*-Curcumene	α-Zingiberene	β-Bisabolene	β-Sesquiphellandrene	*ar*-Turmerone	α-Turmerone	Germacrone	Curlone (= β-Turmerone)	(6*S*,7*R*)-Bisabolone	(*E*)-α-Atlantone
fRh-SD-India (APRC)	2.41	2.44	3.22	0.53	0.89	0.34	0.29	0.98	36.02	16.95	0	19.07	0.81	0.38
Resin-SD-India (APRC)	0.03	0.07	0.10	0	9.75	12.75	2.54	14.59	15.02	17.67	0	9.11	0.17	0.71
Rh-SD-Nepal (APRC)	1.08	0.41	1.17	0.17	3.26	5.97	1.01	7.72	21.20	28.80	0	18.20	1.18	2.68
Rh-SD-Nepal (APRC)	0.43	0.32	1.55	10.22	2.15	5.02	0.87	5.48	20.51	26.73	0	12.99	0.87	1.71
Rh-SD-Indonesia (APRC)	2.94	0.67	0.45	0.57	3.14	2.77	0.75	3.38	14.54	15.41	0	10.03	0	0.90
Rh-CO_2_-India (APRC)	0.41	0.45	0.35	0	3.87	3.35	0.82	4.69	33.82	20.62	0	14.42	1.29	3.26
Rh-CO_2_-India (APRC)	0.28	0.44	1.05	0	5.66	4.20	1.35	8.08	32.02	13.22	0	14.70	1.46	2.59
Rh-CO_2_-India (APRC)	0.61	0.45	1.89	0	4.41	6.29	1.49	9.15	25.23	19.96	0	14.63	1.16	2.02
Rh-SD-Indonesia (APRC)	3.14	0.66	0.77	0.50	1.30	1.80	0.30	1.95	21.51	27.90	0	17.65	1.17	1.90
Rh-SD-Indonesia (APRC)	3.10	1.19	1.15	0.76	11.07	3.23	2.16	8.44	17.45	13.17	0	8.81	0.62	0.72
Rh-HD-Jamaica (APRC)	2.20	0.70	1.77	0.66	1.28	1.76	0.35	1.76	22.19	34.24	0	16.40	0.74	0.30
Rh-Unknown (APRC)	0.45	0.26	1.14	0.30	2.46	4.94	0.79	4.71	25.32	25.30	0	17.49	0.88	1.04
Rh-SD-India (APRC)	0.31	0.47	1.44	0	5.50	4.71	1.46	7.77	32.12	15.06	0	13.18	1.27	2.11
Rh-Unknown (APRC)	1.25	1.40	4.04	0	6.59	6.12	1.57	9.51	29.00	13.38	0	11.97	1.07	1.50
Rh-SD-Indonesia (APRC)	1.58	0.50	0.91	0.32	2.05	2.41	0.43	2.39	21.95	31.05	0	18.86	0.71	1.09
Rh-SD-Nepal (APRC)	3.79	2.52	2.24	1.52	4.09	6.08	1.15	3.20	21.84	20.21	0	9.72	0.38	1.63
Rh-SD-Nepal (APRC)	0.05	0	0.23	1.58	0.99	8.81	0.94	5.65	12.52	44.51	0	14.44	1.06	0.26
dRh-SD-India (APRC)	3.36	2.31	0.79	0.33	2.62	1.42	0.44	2.03	36.64	23.73	0	15.74	0.66	0.19
fRh-SD-India (APRC)	1.13	0.44	0.35	0.21	2.65	3.64	0.74	2.94	28.77	26.50	0	13.68	0.61	2.31
Rh-SD-Nepal (APRC)	0	0.10	0.26	1.11	1.12	0.48	0.18	1.13	36.37	12.57	10.29	12.22	1.10	0.58
Rh-HD-Nepal (APRC)	0.03	0.09	0.38	1.54	0.87	0.54	0	1.06	35.07	20.50	11.11	14.18	0.95	0.26
Rh-Unknown (APRC)	0.05	0.03	0.20	1.54	0.95	8.58	0.83	5.51	12.19	43.30	0	14.11	1.03	0.21
Rh-Unknown (APRC)	6.73	0.79	1.49	0.41	1.70	3.30	0.43	2.72	18.20	37.75	0	37.75	0.40	0.64
Rh-SD-Nepal (APRC)	0.16	0.09	0.30	4.38	1.69	4.28	0.68	4.73	23.18	28.93	0	14.76	1.13	2.38
Rh-CO_2_-India (APRC)	0.36	0.52	1.26	0	5.65	4.13	1.25	8.00	35.08	13.67	0	15.06	1.31	2.49
Rh-CO_2_-India (APRC)	0	0	0.3	0	3.13	2.78	0.76	4.43	34.20	21.49	0	16.34	0	3.77
Rh-SD-Nepal (APRC)	0.16	0.16	0.18	6.04	0	2.56	0.27	2.15	27.36	32.11	0.92	16.72	1.27	0.63
dRh-SD-India (APRC)	1.13	0.98	1.20	8.91	6.14	5.98	1.50	3.17	32.16	9.39	0	3.96	0	0
Rh-HD-Nepal (APRC)	0	0.07	0.39	1.54	0.90	0.66	0.14	1.07	34.42	20.25	11.10	13.90	0.98	0.29
Rh-HD-Nepal (APRC)	0.01	0.01	0.13	0.76	1.71	4.53	0.70	4.04	23.68	35.42	0	14.43	1.04	0.28
Rh-HD-Nepal (APRC)	0.41	0.30	1.51	10.01	2.10	4.90	0.82	5.35	20.12	26.20	0	12.72	0.83	1.67
Rh-HD-Nepal (APRC)	0.03	0.08	0.49	2.87	1.29	1.87	0.37	2.67	31.45	26.92	0	15.65	1.14	0.36

Rh = rhizome; dRh = dried rhizome; fRh = fresh rhizome; HD = hydrodistillation; SD = steam distillation; CO_2_ = supercritical CO_2_ extracts; APRC = from the collection of the Aromatic Plant Research Center.

**Table 2:** Chemical composition of *Curcuma aromatica* rhizome essential oils.

Compound	Car India [15]	Car India [42]	Car India [42]	Car Thailand [38]	Car Japan [37]	Car Japan [37]	Car India [37]	Car India [39]	Car India [39]	Car India [40]	Car India [41]
α-Pinene	1.5	5.9	5.7	0.5	0.4	0.9	0.2	0.4	0.3	0.3	0.8
Camphene	10.2	0.9	1.1	2.0	0	0	0.3	0.9	0.8	0.7	7.4
Myrcene	1.2	0	0	0.4	0.1	0.3	0.1	0.2	0.2	0.2	1.0
1,8-Cineole	10.1	13.7	15.9	0.3	7.5	25.3	1.0	0.1	0.1	5.5	9.3
Terpinolene	0	5.2	3.9	0	0	0	0	tr	tr	0	0.1
Linalool	2.1	0	0	0.6	2.2	2.8	0.1	0	0	0.2	1.2
Camphor	18.8	48.3	45.7	26.9	0	0	3.9	3.9	3.3	32.3	25.6
Isoborneol	1.8	12.2	10.1	2.3	0	0	0.3	0	0	3.4	8.2
Borneol	8.2	5.0	4.9	1.7	0	0	0.3	1.8	1.1	1.1	2.5
α-Terpineol	0	0	0	0	0.4	1.3	1.4	0	tr	0.6	1.0
β-Elemene	7.5	0	0	0.1	4.0	2.5	1.0	0.2	0.2	1.4	1.4
β-Caryophyllene	2.0	0	0	0	1.9	1.7	0.3	0	0	0.3	0.3
α-Humulene	0	0	0	1.9	2.1	1.0	0	0	0	tr	tr
β-Farnesene	0	0	0	0	0	0	2.6	0	0	tr	0
ar-Curcumene	0	0	0	23.2	0	0	22.1	23.6	24.1	3.1	0
Germacrene D	1.8	0	0	0	1.1	0.7	0.2	tr	0.3	0	0.9
Curzerene	0	0.3	0.4	1.4	0	0	3.2	4.6	6.0	0.2	2.7
β-Curcumene	0	0	0	3.9	0	0	29.9	28.4	31.4	0	0
Germacrene B	2.8	0.2	0.4	0.9	0	0	0	0	0	0.3	0.4
Caryophyllene oxide	0	0	0	0	1.4	2.0	0	0	0	tr	tr
Curzerenone	0	0	0	3.8	0	0	3.6	7.3	5.5	11.0	10.9
Germacrone	0	0.3	0.3	0.3	9.6	4.6	4.9	3.6	6.1	0.5	10.6
Xanthorrhizol	4.8	0	0	18.7	0	0	16.2	8.0	5.3	0	0
Curdione	8	4.8	6.8	0	44.0	32.2	0	0	0	0	0

tr = “trace” (<0.05%)

**Table 3:** Chemical composition of *Curcuma zedoaria* rhizome essential oils.

Compound	Cz Nepal-1 (APRC)	Cz Nepal-2 (APRC)	Cz India (APRC)	Cz India [15]	Cz India [43]
1,8-Cineole	8.77	38.39	7.00	0	1.9
Camphor	8.79	0	8.26	3.3	10.3
Borneol/Isoborneol	1.81	0.07	3.17	0.2	2.7
α-Terpineol	1.49	1.17	0.47	1.7	0.3
α-Terpinyl acetate	2.29	0	0	0	0
α-Copaene	17.35	0.42	0	0	0
β-Elemene	2.89	0.21	5.54	0.3	tr
β-Caryophyllene	8.28	1.37	1.46	0	0.4
γ-Elemene	0.29	0	0.84	2.5	0.1
*ar*-Curcumene	0	0.51	0	12.1	0
Zingiberene	0	0	0	12.0	0
Curzerene	2.36	0	5.93	8.0	0
α-Farnesene	0	0	0	2.3	0
γ-Cadinene	0	2.20	0	0	0
δ-Cadinene	3.83	3.85	0.25	0	0
Germacrene B	0.38	0	1.08	6.0	0.6
β-Sesquiphellandrene	0	0	0	9.8	0
Elemol	0	6.84	0	0	0
Curzerenone/*epi*-Curzerenone	20.89	0	29.41	19.0	31.6
Germacrone	2.59	0	6.65	0	10.8
Curlone (= β-Turmerone)	0	0	0	4.0	0
Curdione	0.10	0	1.23	0	1.3
Curcumenol	0	0	1.57	0	2.2

**Table 4:** Chemical composition of *Curcuma aeruginosa* rhizome essential oils reported in the literature.

Compound	Cae Thailand [4]	Cae India [15]	Cae Thailand [38]	Cae Malaysia [46]	Cae Malaysia [45]
Camphene	1.2	0.18	0.3	1.6	0.2
β-Pinene	0.4	27.5	7.7	1.6	0.4
1,8-Cineole	2.7	0.42	9.6	25.2	11.2
Camphor	29.4	0	0	6.8	10.5
Isoborneol	7.3	0	0.6	1.5	3.2
Borneol	2.9	0	0.5	0.5	1.3
β-Elemene	1.4	0	0.2	1.7	2.2
β-Farnesene	0	1.5	0	0.5	1.0
Zingiberene	0	1.2	0	0.1	0
Curzerene	4.8	0	1.1	0	0
Germacrene B	5.2	0	0.5	0	0
Curzerenone	0	0	41.6	30.4	24.6
β-Eudesmol	0	3.6	0	0	0
Germacrone	21.2	0	1.0	2.8	2.7
Curcumenol	0	38.7	0	0	5.6

**Table 5:** Chemical composition of *Curcuma longa* rhizome volatile oils from the published literature.

Sample	α-Phellandrene	*p*-Cymene	1,8-Cineole	Terpinolene	*ar*-Curcumene	α-Zingiberene	β-Bisabolene	β-Sesquiphellandrene	*ar*-Turmerone	α-Turmerone	Germacrone	Curlone (= β-Turmerone)	(6*S*,7*R*)-Bisabolone	(*E*)-α-Atlantone
fRh-HD-Nigeria [47]	15.5	2.1	10.3	3.2	0.7	2.0	0	1.8	10.0	35.9	0	12.9	0	0
dRh-HD-China [48]	0	0.5	0.5	0.3	6.1	20.1	5.1	15.5	27.5	0.1	0.3	1.7	0	0
fRh-HD-India [15]	3.1	0.3	0.7	0.1	3.5	4.0	0	0.8	49.8	9.1	0	7.9	0	0
dRh-HD-Iran [49]	2.2	0.4	0.4	1.5	0.8	1.5	0.4	1.3	68.9	20.9	0	0	0	0
fRh-HD-India [50]	0.1	0.3	0.4	2.7	1.6	2.5	0.8	2.9	24.4	20.5	1.0	11.1	1.7	0.9
dRh-HD-India [50]	0	0.1	0.1	tr	6.6	0.8	4.1	4.2	21.4	0.6	2.6	4.3	0.8	2.6
fRh-HD-India [51]	2.0	0.6	0.8	0.2	1.9	2.6	0.4	2.4	21.0	33.5	0	18.9	0	0
dRh-HD-India [51]	tr	0	tr	0	1.2	2.2	1.5	2.8	30.3	26.5	0	19.1	0	0
fRh-HD-India [52]	8.0	4.3	11.2	0.7	4.4	5.6	2.8	7.1	7.3	11.1	0.1	5.0	0.1	0.2
fRh-HD-India [53]	9.4	1.2	1.9	1.2	0.5	2.3	0	1.8	5.4	44.1	0.4	18.5	0	1.1
fRh-HD-India [54]	0.1	0.1	2.6	0.1	0.2	1.3	0.2	0	31.7	12.9	0.9	12.0	0.2	1.5
dRh-HD-India [55]	2.2	1.0	0	0	4.8	0	0	0	53.1	6.2	0	6.4	0	0
dRh-HD-Thailand [49]	2.2	0.4	0.4	1.5	0.8	1.5	0.4	1.3	68.9	20.9	0	0	0	0
fRh-HD-Pakistan [56]	0.4	0	1.6	0	0	0	0	0	25.3	18.4	0	12.5	0	0
fRh-HD-Bangladesh [57]	0.5	0.2	0	0	3.3	4.4	0.2	5.6	27.8	17.2	0	13.8	0	0
fRh-SD-Bhutan [58]	1.7	0.5	7.6	0.7	1.4	4.2	0.7	3.6	16.7	30.1	0	14.7	1.0	1.2
fRh-HD-Brazil [59]	6.5	0.9	3.2	1.4	1.0	1.9	0.3	1.4	12.9	42.6	0.5	16.0	0.3	0.5
dRh-HD-Brazil [60]	1.7	0.8	0.7	0	2.6	1.0	0	2.4	33.2	23.5	0	22.7	3.1	1.4
dRh-HD-S. Tomé e Principe [35]	15.5	2.5	10.2	3.1	0.8	1.1	0	1.0	12.8	23.9	0	11.5	0	0.6
dRh-HD-S. Tomé e Principe [35]	30.4	5.5	23.0	4.5	1.1	2.4	0	2.0	4.0	12.2	0	4.3	0	0
dRh-HD-Brazil [33]	2.7	0	1.4	0	1.0	2.4	tr	1.9	18.0	44.0	0	18.3	0.6	0.6
fRh-SD-Reunion [61]	1	0.6	2	15.8	4.5	11.8	1.9	8.8	7.7	21.4	0	7.1	0	0
fRh-HD-India [62]	5.3	0	2.6	0	3.5	0	0.6	1.7	49.1	0	0	16.8	0	0
dRh-HD-India [63]	1.8	1.3	1.3	0	1.4	1.7	0	1.7	34.0	34.0	0	15.0	0	0
dRh-HD-India [63]	1.4	0.9	1.3	0	1.5	1.9	0	1.9	35.0	35.0	0	12.0	0	0
Rh-CO_2_-Brazil [64]	4.1	1.5	4.0	1.3	3.6	6.4	1.7	7.7	15.5	20.3	0	15.6	0.3	0.6
dRh-CO_2_-China [65]	0	0	0	2.2	1.9	16.9	1.5	10.0	11.0	40.8	0	14.1	0	0

Rh = rhizome; dRh = dried rhizome; fRh = fresh rhizome; HD = hydrodistillation; SD = steam distillation; CO_2_ = supercritical CO_2_ extracts.

**Table 6:** Concentration (%) of centroids used in the cluster analysis of *Curcuma longa* rhizome oils.

Compound	Cluster 1	Cluster 2	Cluster 3	Cluster 4
α-Phellandrene	6.58	0.71	2.13	2.99
1,8-Cineole	5.11	1.11	1.39	0.82
*ar*-Curcumene	4.77	2.72	1.49	2.68
α-Zingiberene	6.23	2.72	4.68	1.4
β-Sesquiphellandrene	6.22	3.89	3.92	1.02
*ar*-Turmerone	15.94	31.68	18.31	57.96
α-Turmerone	15.49	20.56	35.11	11.41
Curlone	8.01	14.75	17.20	6.22

**Table 7:** Concentration (%) of centroids used in the cluster analysis of *Curcuma aromatica* rhizome oils.

Compound	Cluster 1	Cluster 2	Cluster 3
Camphor	28.28	0	3.69
*ar*-Curcumene	8.76	0	23.25
Curdione	0	38.08	0
β-Curcumene	1.30	0	29.93
1,8-Cineole	5.02	16.41	0.38
Xanthorrhizol	6.23	0	9.83
Curzerenone	8.57	0	5.43
Germacrone	3.80	7.09	4.85

**Table 8:** Concentration (%) of centroids used in the cluster analysis of *Curcuma aromatica* rhizome oils.

	Cluster 1	Cluster 2
Curzerenone/*epi*-Curzerenone	27.3	0
1,8-Cineole	4.42	38.39
Camphor	7.66	0
Germacrone	5.01	0
α-Copaene	4.43	0.42
Curzerene	4.07	0
*ar*-Curcumene	3.03	0.51
Zingiberene	3.00	0
β-Sesquiphellandrene	2.45	0

**Table 9:** Concentration (%) of centroids used in the cluster analysis of *Curcuma aeruginosa* rhizome oils.

	Cluster 1	Cluster 2	Cluster 3
Curzerenone	0	0	32.21
1,8-Cineole	2.68	0.42	15.35
Camphor	29.39	0	5.77
Curcumenol	0	38.70	1.87
β-Pinene	0.35	27.50	3.24
Germacrone	21.21	0	2.16
Isoborneol	7.27	0	1.76
Curzerene	4.84	0	0.36
Germacrene B	5.20	0	0.17
